# Quality of life related to health in adolescents with type 1 diabetes

**DOI:** 10.1186/1758-5996-7-S1-A180

**Published:** 2015-11-11

**Authors:** Ana Clara Barreto Marini, Camila Rodrigues da Cunha Santos, Jéssika Martins Siqueira, Rosana de Morais Borges Marques

**Affiliations:** 1 Universidade Federal de Goiás, Goiânia, Brazil

## Background

The knowledge of the influence of type 1 diabetes on quality of life (QL) of adolescents is important due to the intensive care imposed by the treatment that requires changes in their routine. Therefore, identify the QL contributes to comprehension of the health state and the difficulties of adolescents that are essential for planning measures to improve adherence to treatment. Objective: To evaluate the quality of life related to health of adolescents with type 1 diabetes (T1D).

## Materials and methods

A cross-sectional study was conducted in June 2015 (CEP 078/2011) with adolescents aged 10 to 18, diagnosed with T1D for over a year. We collected socioeconomic, demographic, health status, insulin therapy and metabolic control data. The instrument used to measure quality of life was IQVJD (NOVATO, 2008). The data were analyzed by SPSS version 18.0.

## Results

The study included 53 adolescents (52.8% female; 37.8% aged 16-18 yrs.), most was attending the middle school (52.8%). About sanitation and income conditions, 15.1% and 54.7% didn't have treated water supply and sewerage, respectively, and 36.0% had monthly per capita income less than two minimum wages. 62.7% of the adolescents were diagnosed with T1D before complete 10 yrs. of age. Almost all reported making home glucose monitoring (96.2%), 41.5% for at least three times per day. Complications of diabetes were found in 11.3% of participants, and the median of HbA1c was 9.7% (6-20) and average of 251.50 mg/dL for fasting glucose. The predominant insulin regimen was the intensive with multiple doses, and only 53.9% did rotation of application sites. Regarding nutritional status, 21.2% and 23.5% were overweight and in cardiovascular risk, respectively. The analysis of IQVJD demonstrated that 77.4% were classified as improved QL. As for the domain satisfaction, only 67.9% had better QL, and in the areas impact and concern, 73.6% and 62.3% showed better QL, respectively. We observed that only 47.2% reported good health compared with other teens at the same age.

## Conclusions

The high prevalence of adolescents with poorer QL in the areas satisfaction and concern, as well as in the self-perception of their health, may indicate that the presence of disease and health care that is required, influence negatively in adolescent life, which could affect their adherence to treatment and increase the risk of developing chronic complications.

